# Estimating the impact of body mass index on bladder cancer risk: Stratification by smoking status

**DOI:** 10.1038/s41598-018-19531-7

**Published:** 2018-01-17

**Authors:** Jin Bong Choi, Eun Joo Lee, Kyung-Do Han, Sung-Hoo Hong, U-Syn Ha

**Affiliations:** 1 Department of Urology, Bucheon St. Mary’s Hospital, College of Medicine, The Catholic University of Korea, Bucheon, Republic of Korea; 2 Department of Big Data Steering, National Health Insurance Service, Wonju, Republic of Korea; 3 Department of Biostatistics, College of Medicine, The Catholic University of Korea, Seoul, Republic of Korea; 4 Department of Urology, Seoul St. Mary’s Hospital, College of Medicine, The Catholic University of Korea, Seoul, Republic of Korea; 5 The Cancer Research Institute, The Catholic University of Korea, Seoul, Republic of Korea

## Abstract

We estimated the impact of obesity on bladder cancer with stratification by smoking status using nationally representative data on the Korean population from the National Health Insurance System (NHIS). Of the 45,850,458 people who underwent at last one health examination from 2009 to 2012, 23,378,895 without bladder cancer were followed from the January 2009 to the December 2015. First, the HR for bladder cancer was lowest in people with a BMI < 18.5 (HR = 0.92) and highest for those with BMI ≥ 30 (HR = 1.17) in multiple Cox regression analyses. The positive association between bladder cancer and BMI showed an increasing trend beyond the reference BMI. Second, an analysis of HR for bladder cancer stratified by obesity across smoking status strata showed a significant trend of increasing HR for bladder cancer across obesity and smoking status in multivariate-adjusted models. Conclusively, this population-based study showed that increasing BMI was a risk factor for bladder cancer independent of confounding variables. When stratified by smoking status, there was still a positive association between bladder cancer and BMI (P for trend < 0.01).

## Introduction

Bladder cancer is the ninth most common cancer worldwide, with an estimated 430,000 new cases in 2012. More than 60% of all bladder cancer cases and half of all 165,000 bladder cancer deaths occur in less-developed regions of the world^1^. An annual survey of cancer statistics in Korea in 2012 reported 2,798 new cases of bladder cancer in men and 687 new cases in women. The disease ranked as the ninth most common cancer among men. The estimated crude rate was 11.1% and the age-standardized rate was 8.3%^2^.

Among several risk factors for bladder cancer, smoking is responsible for about half of cases^3,4^. Other proven risk factors for bladder cancer such as occupational exposure to aromatic amines and alcohol drinking are major causes in selected populations^5–7^.

For bladder cancer, epidemiological studies report inconsistent associations between body mass index (BMI) and bladder cancer risk^8–12^. This lack of consistency may be attributable to uncontrolled potential confounders. Among potential confounders, smoking is one of the important controllable risk factors. In many studies, smoking status was not analyzed separately, but considered only as an adjusting factor. Song *et al*. argue that estimates of influence for excess body weight on cancer risk should be given separately for smokers and nonsmokers^13^.

Previous studies on body weight and cancer risk were primarily based on Western populations. Compared to Western populations, Asian populations of the same age generally have more accumulated body fat and higher levels of adipocytokines at equivalent BMI levels^14^. Accordingly, to evaluate the influence of body weight on developing bladder cancer for Asian people, an Asian population is essential.

Therefore, we analyzed the association between obesity and bladder cancer using nationally representative data on the Korean population. Furthermore, we assessed the influence of obesity on the development of bladder cancer with stratification by smoking status.

## Materials and Methods

### Data source and study population

We used the national health insurance claims database established by the National Health Insurance Service (NHIS) of Korea^15^. In Korea, almost all residents are enrolled in the NHIS as an employee or a member of the community. The NHIS contains comprehensive health-related information for approximately 97% of the Korean population in eligibility, health examination, medical treatment and medical care institution databases. The remaining 3% of the population with low income is covered by the Medical Aid program. Information on Medical Aid beneficiaries has been integrated into the NHIS database since 2006. Therefore, data extracted from the NHIS database are considered to represent the entire Korean population^16^.

In this study, age, sex, and diagnostic codes based on the International Classification of Diseases, Tenth Revision, Clinical Modification (ICD-10-CM) were retrieved. Bladder cancer was coded C67. Because the Korean government enhanced benefit coverage for four major conditions (cardiovascular disease, cerebrovascular disease, cancer and rare diseases), all physician-diagnosed cancer patients are registered in the database^17^. Patient identification numbers were anonymized to protect individual privacy. Anonymized and de-identified information was used for analysis, and therefore the need for informed consent was waived.

**Figure 1:**
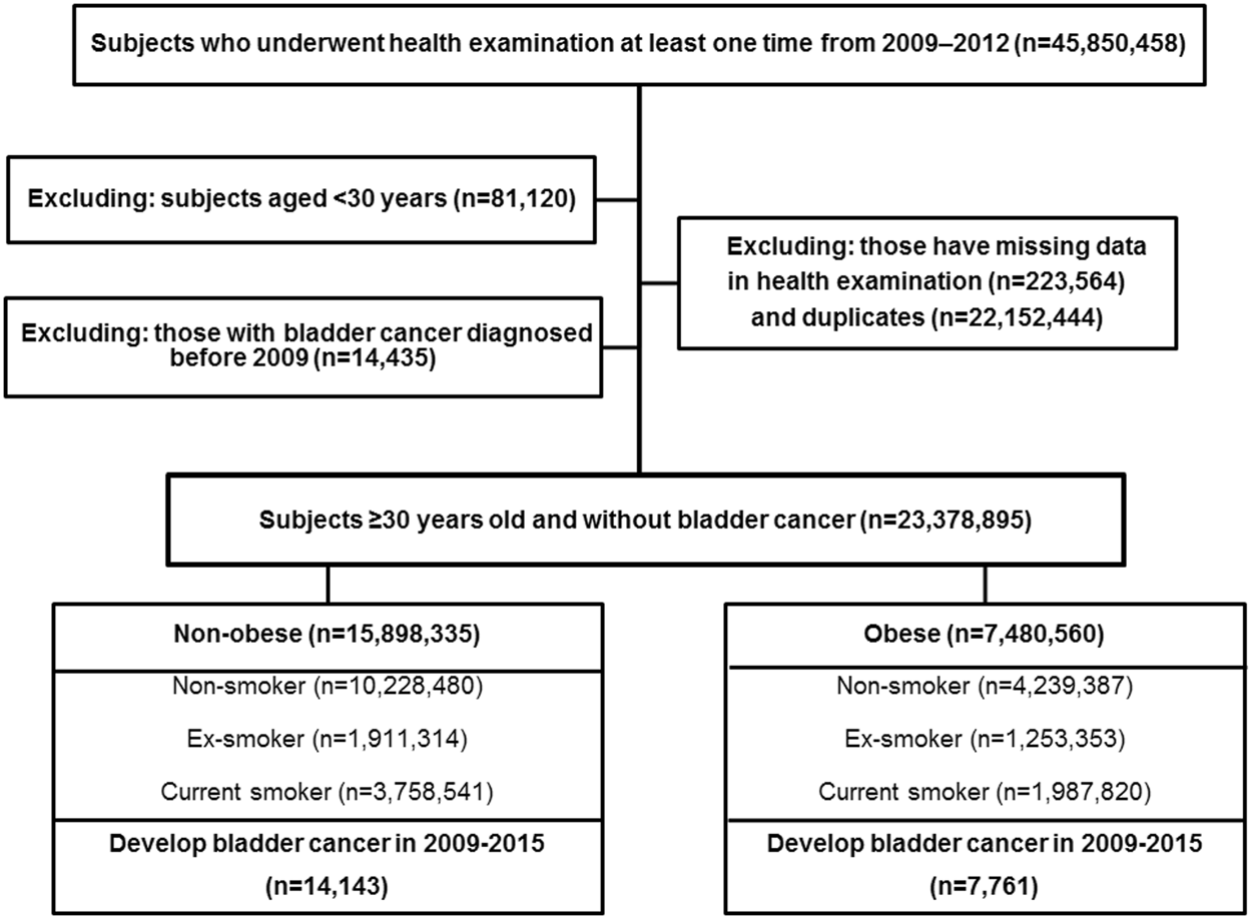
Study design and disposition of participants.

Of the 45,850,458 people who underwent at least one health examination from 2009–2012, those aged <30 years (n = 81,120) were excluded because bladder cancer is rare in this population. The information about smoking status and BMI were reported in health examinations. We analyzed data stratified into ages 30–39, 40–64 and ≥65 years according to incidence rate. After excluding people with missing data for health examinations (n = 223,564), duplicates (n = 2,212,444) and people with bladder cancer diagnosed before 2009 (n = 14,435), 23,378,895 individuals without bladder cancer were followed from the January 2009 to the December 2015. A flowchart for selecting cases for the study is in Fig. 1. This study was approved by the Institutional Review Board of the Catholic University of Korea (No. KC16RISI0944). All methods were performed in accordance with the approved guideline and regulation.

### Demographic factors

BMI was calculated as weight in kilograms divided by height in square meters. The Korean Society for the Study of Obesity recommends using BMI categories of underweight (under 18.5), normal weight (18.5 to 22.9), overweight (23 to 24.9), obese (25 to 29.9), and severely obese (over 30)^18^. Diabetes was identified by diagnostic codes (E10–14) from the ICD-10-CM for self-reported medical history of diabetes or fasting serum glucose level ≥126 mg/dl in the health examination database. Hypertension was defined as previous hypertension diagnosis (I10–13, I15), history of taking at least one antihypertensive drug, or as blood pressure ≥140/90 mmHg in the health examination database. Dyslipidemia was identified by diagnostic codes E78, self-reported use of lipid-lowering drugs, or a total cholesterol level ≥240 mg/dl in the health examination database.

For lifestyle variables, smoking status was categorized into three groups: nonsmokers, current smokers who smoked 100 cigarettes or more in their lifetime, and ex-smokers who had smoked in the past but had quit. Alcohol consumption status was categorized into three groups: ‘never drinkers,’ ‘up to 30 g/day’ who drank up to an average of 30 g of alcohol a day, and ‘more than 30 g/day.’ People were categorized as doing exercise regularly considering the answer to the question, “During the last week, how many days did you exercise vigorously for over 20 minutes until you were almost out of breath?”

### Statistical analysis

SAS version 9.4 (SAS Institute Inc., Cary, NC, USA) was used for statistical analysis. Data were presented as proportion for continuous or categorical variables or mean ± standard deviation (SD). Multiple Cox regression analysis was conducted to examine hazard ratios (HRs) and 95% confidence intervals (CIs) for associations between bladder cancer and obesity with stratification by smoking status. Calculations were adjusted for age, alcohol consumption, and regular exercise^19,20^. P for trend which used to test a linear trend of the HRs was a value less than 0.05 was considered statistically significant. A p-value < 0.05 was also considered statistically significant.

## Results

During 126,318,472.4 person-years of follow-up, 21,904 incident cases of bladder cancer developed between the beginning of 2009 and end of 2015. Incidence density was 17.3 cases per 100,000 person-years.

### General characteristics according to obesity/nonobesity and smoking status

General characteristics of the study population and subgroups are in Table 1. Among the total 23,378,895 individuals, 14,143 (0.09%) in the nonobesity group and 7,761 (0.1%) in the obesity group were diagnosed with bladder cancer. An estimated 27% of the population diagnosed with bladder cancer was categorized as overweight and approximately 35% was categorized as obese. The obesity group was more likely to have diabetes, hypertension, and dyslipidemia. Ex-smokers were more likely to have diabetes, hypertension, and dyslipidemia. According to smoking status, diagnoses of bladder cancer were 9,077 (0.06%) in the never-smoking, 5,843 (0.18%) in the ex-smoking, and 6,984 (0.12%) in the current-smoking groups.

**Table 1:** General characteristics according to obesity and nonobesity or smoking status.

	BMI		Smoking status		
	Nonobesity	Obesity	Non	Ex	Current
No. in population	15,898,335	7,480,560	14,467,867	3,164,667	5,746,361
No. of diagnosed bladder cancers	14,143	7,761	9,077	5,843	6,984
Age, years	46.79 ± 14.69	49.53 ± 13.57	48.87 ± 14.83	50.03 ± 13.4	43.36 ± 12.88
30–39	5,020,233 (31.58)	1,781,074 (23.81)	3,719,507 (25.71)	707,069 (22.34)	2,374,731 (41.33)
40–64	8,756,176 (55.08)	4,562,842 (61.00)	8,369,611 (57.85)	1,973,733 (62.37)	2,975,674 (51.78)
≥65	2,121,92 (13.35)	1,136,644 (15.19)	2,378,749 (16.44)	483,865 (15.29)	395,956 (6.89)
Sex, male	7,447,143 (46.84)	4,410,362 (58.96)	3,653,008 (25.25)	2,927,507 (92.51)	5,276,990 (91.83)
BMI, kg/m^2^	21.94 ± 2.00	27.4 ± 2.19	23.45 ± 3.31	24.35 ± 2.98	23.91 ± 3.28
Smoking status					
Non	10,228,480 (64.34)	4,239,387 (56.67)			
Ex	1,911,314 (12.02)	1,253,353 (16.75)			
Current	3,758,541 (23.64)	1,987,820 (26.57)			
Alcohol consumption					
Never drinkers	8,602,269 (54.68)	3,810,261 (51.45)	10,162,513 (69.93)	1,000,822 (32.03)	1,398,616 (24.57)
Up to 30 g/day	6,228,699 (39.60)	2,962,311 (40.00)	4,034,602 (28.18)	1,759,881 (56.33)	3,396,527 (59.66)
More than 30 g/day	900,054 (5.72)	632,561 (8.54)	270,752 (1.89)	363,603 (11.64)	898,260 (15.78)
Regularly exercise	7,719,193 (48.87)	3,809,810 (51.22)	6,588,171 (45.79)	1,946,117 (61.91)	2,994,71 (52.52)
Hypertension	3,205,637 (20.16)	2,955,000 (39.50)	3,794,070 (26.22)	1,060,328 (33.51)	1,306,239 (22.73)
Dyslipidemia	2,434,557 (15.31)	2,068,306 (27.65)	2,856,954 (19.75)	687,905 (21.74)	958,004 (16.67)
Diabetes	1,136,109 (7.15)	1,038,742 (13.89)	1,237,105 (8.55)	393,764 (12.44)	543,982 (9.47)

Data are presented as the mean ± SD, or % (SD).

BMI: body mass index, SD: standard deviation.

### Increasing trend in risk of bladder cancer according to age, BMI, and smoking status

**Figure 2:**
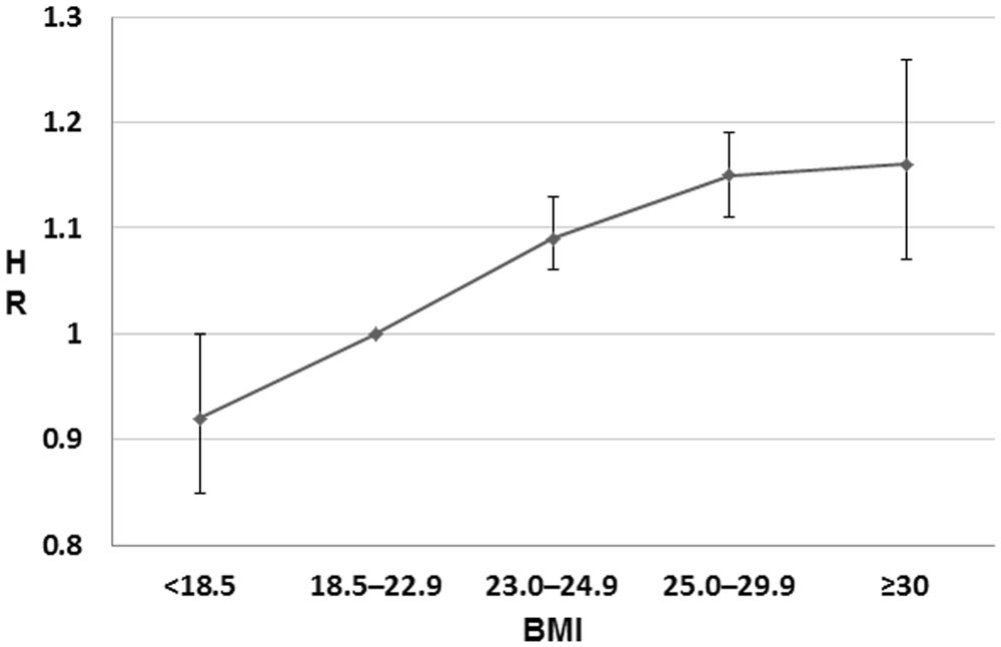
Hazard ratio of bladder cancer according to body mass index. Error bars represent 95% confidence intervals for lower and upper limits. Multivariable adjusted hazard ratio of bladder cancer increased across body mass index.

The results of multiple cox regression analyses for bladder cancer development are shown in Table 2. Increasing age, BMI, smoking status, dyslipidemia, diabetes, and hypertension were risk factors. The HR for bladder cancer was lowest in people with a BMI <18.5 and highest for those with BMI ≥30 in both models. A significant increasing trend in risk of bladder cancer was seen with increasing BMI in a multivariate-adjusted model (Fig. 2). There was also an increasing trend when stratified by sex (Table S1). And association trends for bladder cancer and BMI or smoking status were similar in an age-categorized population (Table S2).

**Table 2:** Multiple cox regression analyses for bladder cancer development.

				H.R (95% confidence interval)	
	Event	Person year	Incidence*	Model 1^†^	Model 2^‡^
Age, years					
30–39	479	36,724,384.94	1.30	Ref.	Ref.
40–64	9,493	72,038,460.09	13.18	10.11 (9.22, 11.08)	10.65 (9.71, 11.69)
≥65	11,932	17,555,627.37	67.97	52.03 (47.49, 57.01)	52.29 (47.58, 57.47)
Sex					
Male	17,979	64,487,512.03	27.88	Ref.	Ref.
Female	3,925	61,830,960.37	6.35	0.23 (0.22, 0.24)	0.20 (0.19, 0.21)
Smoking status					
Non	9,077	78,119,887.32	11.62	Ref.	Ref.
Ex	5,843	17,281,895.63	33.81	2.90 (2.81, 3.00)	1.32 (1.27, 1.37)
Current	6,984	30,916,689.45	22.59	1.95 (1.89, 2.01)	1.52 (1.46, 1.57)
BMI, kg/m^2^					
<18.5	697	4,968,089.15	14.03	0.95 (0.88, 1.02)	0.92 (0.85, 1.00)
18.5–22.9	7,482	50,015,687.14	14.96	Ref.	Ref.
23.0–24.9	5,964	30,796,378.59	19.37	1.08 (1.05, 1.12)	1.09 (1.06, 1.13)
25.0–29.9	7,145	35,933,349.62	19.88	1.13 (1.10, 1.17)	1.15 (1.11, 1.19)
≥30	616	4,604,967.91	13.38	1.16 (1.07, 1.26)	1.17 (1.05, 1.28)
Alcohol consumption					
Never drinkers	11,796	66,884,498.9	17.64	Ref.	Ref.
Up to 30 g/day	8,059	49,695,192.9	16.22	0.92 (0.89, 0.95)	0.82 (0.79, 0.85)
More than 30 g/day	1,800	8,250,025.06	21.82	1.24 (1.18, 1.30)	0.79 (0.75, 0.83)
Regularly Exercise					
No	11,479	62,586,593.14	18.34	Ref.	Ref.
Yes	10,283	62,800,931.27	16.37	0.89 (0.87, 0.92)	0.959 (0.933, 0.986)
Hypertension					
No	10,565	93,064,967.88	11.35	Ref.	Ref.
Yes	11,339	33,253,504.52	34.10	3.00 (2.92, 3.08)	1.29 (1.25, 1.33)
Dyslipidemia					
No	15,700	102,023,111.5	15.39	Ref.	Ref.
Yes	6,204	24,295,360.9	25.54	1.66 (1.61, 1.71)	1.11 (1.08, 1.15)
Diabetes					
No	17,298	114,768,614.4	15.07	Ref.	Ref.
Yes	4,606	11,549,857.98	39.88	2.65 (2.57, 2.74)	1.20 (1.16, 1.25)

*All rates are expressed as number per 100,000 person-years.

^†^Univariate model.

^‡^Full model of multiple cox regression.

### Positive association between obesity and bladder cancer development when stratified by smoking status

HRs for bladder cancer stratified by obesity (BMI ≥25) across strata of smoking status (never, ex and current smoker) are presented in Table 3. There was a positive association between bladder cancer and BMI when stratified by smoking status. The HR for bladder cancer was lowest in nonsmokers with a BMI <18.5 (HR = 0.86) and highest for those with BMI ≥30 (HR = 1.16) in a model adjusted for age, sex, regular exercise, and alcohol consumption (P for trend <0.01). Similar associations between BMI and bladder cancer development were also observed in ex and current smokers (P for trend <0.01).

**Table 3:** Age- and multivariate-adjusted hazard ratios for bladder cancer according to obesity: stratified by smoking status.

BMI, kg/m^2^	Nonsmokers			P for trend	Ex-smokers			P for trend	Current smokers			P for trend
	Event	Incidence*	H.R (95% confidence interval)^†^		Event	Incidence*	H.R (95% confidence interval)^†^		Event	Incidence*	H.R (95% confidence interval)^†^	
<18.5	247	6.86	0.86 (0.76, 0.98)	<0.01	135	24.11	0.93 (0.78, 1.10)	<0.01	314	30.00	0.97 (0.86, 1.09)	<0.01
18.5–22.9	3,043	9.16	Ref.		1,756	33.35	Ref.		2,682	23.26	Ref.	
23.0–24.9	2,464	13.46	1.08 (1.03, 1.14)		1,634	33.69	1.07 (1.01, 1.15)		1,865	24.40	1.14 (1.08, 1.21)	
25.0–29.9	3,015	14.87	1.12 (1.06, 1.77)		2,168	34.80	1.21 (1.33, 1.29)		1,961	20.80	1.17 (1.10, 1.24)	
≥30	308	11.27	1.16 (1.03, 1.31)		152	26.19	1.24 (1.16, 1.31)		162	12.80	1.22 (1.04, 1.43)	

*All rates are expressed as number per 100,000 person-years.

^†^Adjusted for age, sex, regular exercise, and alcohol consumption.

## Discussion

The main findings of this population-based study were: (1) Increasing BMI was a risk factor for bladder cancer independent of confounding variables. (2) The positive association between bladder cancer and BMI showed an increasing trend beyond the reference BMI. (3) There was a positive association between bladder cancer and BMI, even when stratified by smoking status.

Mounting evidence suggests that obesity could be carcinogenic. A study on how obesity might promote carcinogenesis suggested that obesity elevates insulin production, which might drive tumor growth^21^. Accumulation of adipose tissue stimulates the production of pro-inflammatory factors and cytokines (e.g., tumor necrosis factor-α and interleukin-6) and decreases production of the peptide adiponectin. These metabolic abnormalities lead to hyperinsulinism and insulin resistance^21^. These changes can make pancreatic beta cells increase insulin production to maintain normal glucose levels, leading to hyperinsulinemia. Hyperinsulinemia increases the activity of insulin-like growth factor 1^22^, which in turn drives an imbalance in cell proliferation, apoptosis and angiogenesis^21^, influencing the development of bladder cancer^23^.

The other suggestion is that obesity might induce chronic inflammation, which might disrupt the balance between cytokines and pro-inflammatory factors^24^. The concept of obesity has changed so the condition is now considered a chronic subclinical inflammation^25^. The relationship between cancer and inflammation is supported by chronic inflammation-associated cancers such as gastric, cervical, and liver cancer^26^.

Excessive accumulated adipocytes produce leptin, which is a key element in the initiation of the adipose pro-inflammatory cascade^27^. Consequently, inflammatory action in adipose tissue stimulates production of inflammatory cytokines including IL-1β, IL-6, IL-12, and TNFα, and enhances reactive oxygen species production^28^. Chronic inflammation is induced by excessive fat accumulation, which is associated with increased cancer risk.

Many studies found a strong association between smoking and bladder cancer, so smoking is a well-known, important risk for developing bladder cancer^29^. For a biological understanding of the influence on development of bladder cancer, smoking may influence central fat accumulation^30–32^. Consequently, smokers usually have more visceral adipose tissue, but less lean body mass than nonsmokers of the same body weight. In people with a similar BMI, smokers could have a higher fat component than nonsmokers, so the effect of accumulated adipose tissue on bladder cancer risk could be greater in smokers than nonsmokers. This possibility means that smokers would have more severe hyperinsulinemia and chronic inflammation from adipocytes than nonsmokers with a similar BMI.

The distinctive feature of our study is that it is an Asian population-based observational study that estimates the influence of obesity on bladder cancer risk with stratification by smoking status. The study revealed a positive association between bladder cancer and BMI, even when stratified by smoking status. Epidemiological studies report inconsistent associations between BMI and bladder cancer risk. Qin *et al*. reported that obesity is associated with increased risk of bladder cancer through a meta-analysis of cohort studies^8^. Sun *et al*. reported a dose-response meta-analysis of 15 cohort studies that showed a pooled relative risk and corresponding 95% CI of bladder cancer of 1.10 (1.06–1.14) for obesity, with low (I² = 15.5%, P = 0.241) heterogeneity between studies^33^. Among the epidemiologic studies included in this meta-analysis, only one study (Koebnick *et al*., 2008) performed stratified analysis by smoking status. The relative risk for bladder cancer was lowest in current smokers (P trend = 0.502) and highest in never smokers (P trend = 0.208). However, the interaction between obesity and smoking was not also statistically significant (P interaction = 0.862). Song *et al*. suggested that stratifying by smoking status is critical for estimating the influence of obesity on cancer risk. Intractable residual confounding by smoking can occur when assessing cancer risk according to BMI. In people with a similar BMI, nonsmokers are more likely to have relatively higher lean body mass and lower adiposity than smokers. This effect can generate confusion and dilute the effect of obesity on cancer risk^13^. For lung cancer, high BMI is associated with lower risk in overall analysis, but the association disappears when sub-analysis is restricted to never smokers to control for the smoking factor^12,34^. Therefore, stratification by smoking status should provide accurate estimates of the influence of obesity on cancer^13^. Analyses considering smoking status are worthy of notice.

The other distinctive feature of this study is that it used a nationally representative analysis of the Korean population. We analyzed data based on the NHIS database of Korea, which has healthcare information in the entire Korean population registered in the national health insurance service. The data had more than 12 million person-years in the observation period and 21,904 cases of bladder cancer. Sample size and the observation period in this study were, to our knowledge, larger than any previous study for assessment of the impact of obesity and smoking status on bladder cancer. People over 40 years old in South Korea are advised to have a health examination including urine analysis, and if the analysis shows an abnormal finding, the NHIS covers further medical evaluation. Therefore, analysis for bladder cancer in the region where medical checkups including urine analysis is widespread has considerable strength and power.

The current study has several limitations. First, smoking status, a known risk factor for bladder cancer, was evaluated via self-reporting, and was not stratified by smoking amount and duration; smoking could have been misclassified to some extent and might be confounding. Secondly, there was some detection bias. Compared with normal body weight people and non-smokers, obese people and smokers are more likely to visit a hospital for any problems. Thus, doctors are more likely to detect initial symptoms of bladder cancer in such people. This is a problem of nationwide population-based studies using the national health insurance claims database that should be improved in the future. However, many of the subjects underwent health screening including urine analysis every two years. So, the detection bias for the microscopic hematuria that is the most important initial symptom of bladder cancer could be reduced. Furthermore, the inability to distinguish between those who participate in the national health screening program every two years and those who receive a checkup every year through personal insurance is a limitation of research using the national health insurance claims database. Third, some residual confounding factors like social economic status and high risk occupation were not measured and cannot be excluded. So, selection bias could have been an issue in this study. Fourth, BMI can change with time, although our study used single measures of BMI at baseline. So, we could not determine whether reducing fat mass will decrease the risk of bladder cancer. Therefore, future studies should consider the effects of changes in anthropometric measures on bladder cancer risk. Last limitation was that potential disease misclassification could have occurred because we relied on medical claims for information about disease diagnosis. To patients with major diseases such malignancies, NHIS covers almost all medical costs, with patients paying 5% of total medical costs. Therefore, the NHIS has strict criteria for registering and certifying patients with major diseases. Although misclassification can occur for some minor diseases, the NHIS of Korea is sufficiently reliable and trustworthy for medical claims of patients with malignant disease.

## Conclusion

This population-based study showed that increasing BMI was a risk factor for bladder cancer independent of confounding variables. When stratified by smoking status, the positive association between bladder cancer and BMI persisted. Our findings are important for the management of smoking and obesity to prevent bladder cancer and could be a resource to develop public healthcare policy.

## Electronic supplementary material

Supplementary Information
