# Supplementary material for: Estimating the impact of body mass index on bladder cancer risk: Stratification by smoking status
Source: Sci Rep. 2018 Jan 17;8:947. doi: 10.1038/s41598-018-19531-7 (PMC5772621; doi:10.1038/s41598-018-19531-7)
Supplement: Supplementary file 1 — Supplementary Information [file 41598_2018_19531_MOESM1_ESM.doc]

**Estimating the impact of body mass index on bladder cancer risk: Stratification by smoking status**

Jin Bong Choi^1*^, Eun Joo Lee^2*^, Kyung-Do Han^3^, Sung-Hoo Hong^4,5^, and U-Syn Ha^4,5^

1. Department of Urology, Bucheon St. Mary’s Hospital, College of Medicine, The Catholic University of Korea, Bucheon, Republic of Korea. 2. Department of Big Data Steering, National Health Insurance Service, Wonju, Republic of Korea. 3. Department of Biostatistics, College of Medicine, The Catholic University of Korea, Seoul, Republic of Korea. 4. Department of Urology, Seoul St. Mary’s Hospital, College of Medicine, The Catholic University of Korea, Seoul, Republic of Korea. 5. The Cancer Research Institute, The Catholic University of Korea, Seoul, Republic of Korea.

^*^These authors contributed equally to this work. Correspondence and requests for materials should be addressed to U-Syn Ha (E-mail: ushamd@catholic.ac.kr)

**Table S1.** Age- and multivariate-adjusted hazard ratios for bladder cancer according to body mass index and smoking status stratified by men and women.

|  |  |  |  | |  | H.R (95% confidence interval) |
| --- | --- | --- | --- | --- | --- | --- |
| Sex |  | Event | Person year | Incidence^*^ | Model 1^†^ | Model 2^‡^ |
| Men | Smoking status |  |  |  |  |  |
|  | Non | 5,403 | 19,914,558.84 | 27.13 | Ref. | Ref. |
|  | Ex | 5,775 | 16,050,759.7 | 35.98 | 1.35 (1.30, 1.40) | 1.35 (1.30, 1.41) |
|  | Current | 6,801 | 28,522,193.49 | 23.85 | 1.69 (1.63, 1.75) | 1.75 (1.69, 1.82) |
|  | BMI, kg/m^2^ |  |  |  |  |  |
|  | <18.5 | 581 | 1,475,606.85 | 39.37 | 0.97 (0.89, 1.05) | 0.94 (0.86, 1.02) |
|  | 18.5–22.9 | 6,122 | 21,715,183.93 | 28.19 | Ref. | Ref. |
|  | 23.0–24.9 | 4,957 | 17,330,433.67 | 28.60 | 1.09 (1.05, 1.13) | 1.11 (1.06, 1.15) |
|  | 25.0–29.9 | 5,898 | 21,557,737.93 | 27.36 | 1.16 (1.16, 1.20) | 1.19 (1.14, 1.23) |
|  | ≥30 | 421 | 2,408,549.64 | 17.48 | 1.14 (1.03, 1.26) | 1.16 (1.05, 1.29) |
| Women | Smoking status |  |  |  |  |  |
|  | Non | 3,674 | 58,205,328.47 | 6.31 | Ref. | Ref. |
|  | Ex | 68 | 1,231,135.93 | 5.52 | 1.34 (1.06, 1.71) | 1.38 (1.09, 1.76) |
|  | Current | 183 | 2,394,495.96 | 7.64 | 1.67 (1.44, 1.94) | 1.74 (1.50, 2.03) |
|  | BMI, kg/m^2^ |  |  |  |  |  |
|  | <18.5 | 116 | 3,492,482.29 | 3.32 | 0.86 (0.71, 1.04) | 0.85 (0.71, 1.03) |
|  | 18.5–22.9 | 1,360 | 28,300,503.21 | 4.81 | Ref. | Ref. |
|  | 23.0–24.9 | 1,007 | 13,465,944.92 | 7.48 | 1.10 (1.01, 1.20) | 1.10 (1.02, 1.20) |
|  | 25.0–29.9 | 1,247 | 14,375,611.68 | 8.67 | 1.11 (1.03, 1.20) | 1.10 (1.02, 1.19) |
|  | ≥30 | 195 | 2,196,418.27 | 8.88 | 1.27 (1.09, 1.47) | 1.23 (1.05, 1.43) |

^*^All rates are expressed as number per 100,000 person-years.

^†^Adjusted for age

^‡^Adjusted for age, regular exercise, and alcohol consumption

**Table S2.** Age- and multivariate-adjusted hazard ratios for bladder cancer according to body mass index and smoking status in an age-categorized population

|  |  |  |  | | H.R (95% confidence interval) | | | |
| --- | --- | --- | --- | --- | --- | --- | --- | --- |
|  |  | Event | Person-years | Incidence^*^ | Model 1^†^ | P for trend | Model 2^‡^ | P for trend |
| BMI, kg/m^2^ | |  |  |  |  |  |  |  |
| Age, years  29–39 | < 18.5 | 18 | 2,770,428.32 | 0.65 | 0.92 (0.56, 1.50) | < 0.001 | 1.08 (0.66, 1.76) | < 0.001 |
|  | 18.5~22.9 | 171 | 17,158,749.05 | 0.99 | Ref. |  | Ref. |  |
|  | 23.0~24.9 | 104 | 7,144,549.79 | 1.46 | 1.12 (0.88, 1.44) |  | 0.99 (0.77, 1.27) |  |
|  | 25.0~29.9 | 157 | 8,168,996.81 | 1.92 | 1.42 (1.13, 1.77) |  | 1.17 (0.93, 1.46) |  |
|  | ≥ 30 | 29 | 1,481,660.98 | 1.96 | 1.52 (1.02, 2.26) |  | 1.33 (0.89, 1.97) |  |
| Age, years  40–64 | < 18.5 | 193 | 1,559,828.29 | 12.37 | 1.20 (1.03, 1.38) | < 0.001 | 1.11 (0.96, 1.29) | < 0.001 |
|  | 18.5~22.9 | 2,910 | 26,725,016.22 | 10.89 | Ref. |  | Ref. |  |
|  | 23.0~24.9 | 2,663 | 19,073,064.31 | 13.96 | 1.08 (1.03, 1.14) |  | 1.06 (1.01, 1.12) |  |
|  | 25.0~29.9 | 3,417 | 22,154,368.07 | 15.42 | 1.12 (1.06, 1.17) |  | 1.12 (1.06, 1.77) |  |
|  | ≥ 30 | 310 | 2,526,183.19 | 12.27 | 1.05 (0.93, 1.18) |  | 1.13 (1.03, 1.31) |  |
| Age, years  ≥65 | < 18.5 | 486 | 637,832.54 | 76.20 | 1.06 (0.96, 1.16) | < 0.001 | 0.93 (0.85, 1.02) | < 0.001 |
|  | 18.5~22.9 | 4,401 | 6,131,921.87 | 71.77 | Ref. |  | Ref. |  |
|  | 23.0~24.9 | 3,197 | 4,578,764.49 | 69.82 | 1.01 (0.97, 1.05) |  | 1.08 (1.04, 1.14) |  |
|  | 25.0~29.9 | 3,571 | 5,609,984.74 | 63.65 | 1.02 (0.98, 1.07) |  | 1.14 (1.09, 1.19) |  |
|  | ≥ 30 | 277 | 597,123.73 | 46.39 | 1.04 (0.92, 1.18) |  | 1.19 (1.05, 1.35) |  |
| Smoking status | |  |  |  |  |  |  |  |
| Age, years  29–39 | Non | 154 | 19,855,599.06 | 0.78 | Ref. | < 0.001 | Ref. | < 0.001 |
|  | Ex | 76 | 3,924,284.73 | 1.94 | 1.28 (0.95, 1.73) |  | 1.30 (0.96, 1.77) |  |
|  | Current | 249 | 12,944,501.15 | 1.92 | 1.43 (1.13, 1.81) |  | 1.47 (1.15, 1.87) |  |
| Age, years  40–64 | Non | 3,323 | 45,364,549.95 | 7.33 | Ref. | < 0.001 | Ref. | < 0.001 |
|  | Ex | 2,448 | 10,791,373.04 | 22.69 | 1.36 (1.28, 1.45) |  | 1.39 (1.31, 1.48) |  |
|  | Current | 3,722 | 15,882,537.09 | 23.44 | 1.78 (1.68, 1.88) |  | 1.83 (1.73, 1.94) |  |
| Age, years  ≥65 | Non | 5,600 | 12899738.3 | 43.41 | Ref. | < 0.001 | Ref. | < 0.001 |
|  | Ex | 3,319 | 2,566,237.86 | 129.33 | 1.30 (1.24, 1.36) |  | 1.30 (1.24, 1.36) |  |
|  | Current | 3,013 | 2,089,651.21 | 144.19 | 1.59 (1.52, 1.67) |  | 1.65 (1.57, 1.73) |  |

^*^All rates are expressed as number per 100,000 person-years.

^†^Adjusted for age

^‡^Adjusted for age, BMI, smoking status, regular exercise, and alcohol consumption
